# Molecular memory with atomically smooth graphene contacts

**DOI:** 10.1186/1556-276X-8-476

**Published:** 2013-11-14

**Authors:** Ahmad Umair, Tehseen Z Raza, Hassan Raza

**Affiliations:** 1 Department of Electrical and Computer Engineering, University of Iowa, Iowa City, IA 52242, USA; 2 Department of Physics and Astronomy, University of Iowa, Iowa City, IA 52242, USA

**Keywords:** Graphene, Contact, Molecular memory, Write-once read-many, Bucky-ball

## Abstract

We report the use of bilayer graphene as an atomically smooth contact for nanoscale devices. A two-terminal bucky-ball (C_60_) based molecular memory is fabricated with bilayer graphene as a contact on the polycrystalline nickel electrode. Graphene provides an atomically smooth covering over an otherwise rough metal surface. The use of graphene additionally prohibits the electromigration of nickel into the C_60_ layer. The devices exhibit a low-resistance state in the first sweep cycle and irreversibly switch to a high-resistance state at 0.8 to 1.2 V bias. In the subsequent cycles, the devices retain the high-resistance state, thus making it write-once read-many memory.

## Background

Reliable and efficient contacts are an important aspect of device design at the nanoscale level. Historically, the contacts in the micron-scale devices have only been part of the overall device design for minimizing the contact resistance based on Schottky barrier height [1-3]. At the nanoscale level, however, the influence of contacts on the transport channel is so important that an equal or often times even more effort is spent on the contact and interface design [4,5]. In various nanoscale devices, the contacts even dominate the transport characteristics [6,7]. While various novel contacts exist at the nanoscale with unique density of states, the simplest ones are the ohmic contacts used to inject and extract the charge carriers. However, in addition to the atomic roughness and grain boundaries, such contacts suffer from electromigration or filament formation, which may deteriorate the device characteristics and lead to reliability issues [8]. One of the grand challenges thus for the nanoscale design is to provide smooth and reliable contact to nanomaterials, being free from electromigration and any other non-ideal effects. In this paper, our objective is to explore graphene [9,10] nanomembranes as a candidate for such contacts. The use of graphene and boron nitride has been explored earlier for ultrathin circuitry [11].

In this work, we report the use of bilayer graphene (BLG) as an atomically smooth contact in a molecular memory. Although various device structures based on graphene have been explored [12], our study is unique in the context of its use to improve reliability. BLG may prevent the electromigration of Ni atoms into the active material of the device. Furthermore, the use of BLG instead of monolayer or several-layer graphene is twofold. As compared to the monolayer, the probability of complete coverage with BLG is higher in the presence of defects. On the other hand, with the increasing number of layers, the transport properties of the device may be dominated by the multilayer graphene itself. Thus, BLG tends to provide an optimum trade-off.

## Methods

**Figure 1:**
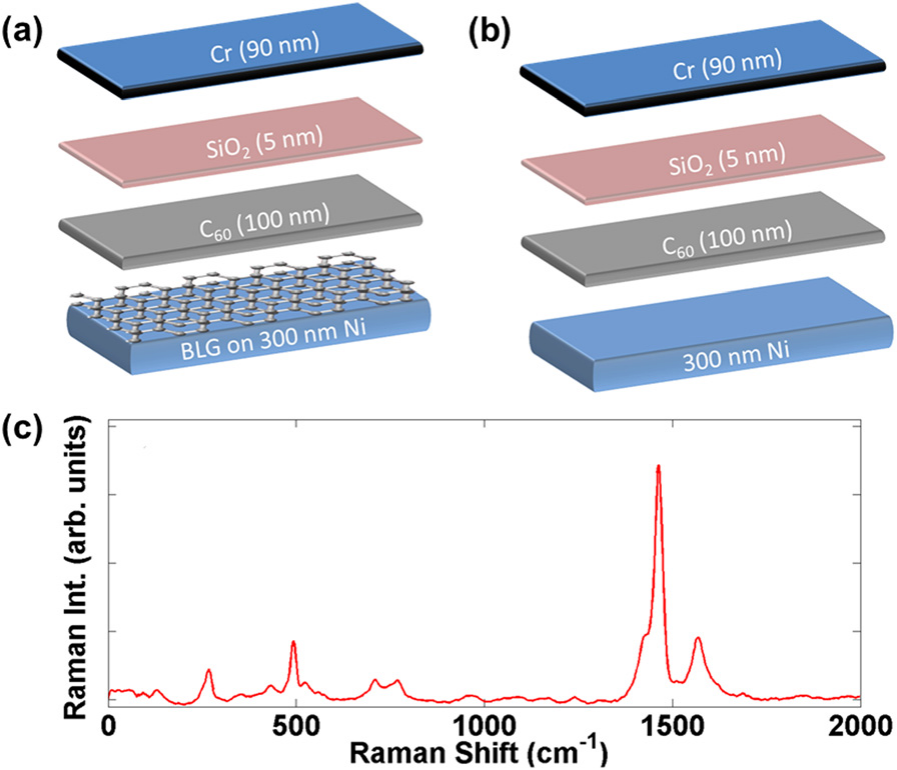
**Device schematics and characterization. (a)** Molecular memory with atomically smooth bilayer graphene sandwiched between 300 nm Ni and 100 nm C_60_ films. **(b)** Control device without the bilayer graphene. **(c)** Raman spectrum of evaporated C_60_ film on the bilayer graphene is shown as well.

The device schematic with BLG contact is shown in Figure 1a. We synthesized BLG on a 300 nm Ni film deposited on a 300-nm thermally grown oxide on Si substrate. Ni was deposited by using electron-beam evaporator (Angstrom Engineering, Kitchener, Ontario, Canada) at 1 Å/s rate under < 7 × 10^−7^ Torr chamber pressure. Ni pallets were placed in an alumina boat (both supplied by International Advanced Materials, Spring Valley, NY, USA) to avoid any contamination or residues. Prior to Ni evaporation, Si/SiO_2_ substrate was cleaned with acetone for 10 min, methanol for 10 min, deionized (DI) water rinse for 10 min, then nanostrip for 20 min (commercial Piranha substitute), followed by DI water rinse for another 10 min. This sequence removes the impurities from the SiO_2_ surface and provides better Ni adhesion. After Ni evaporation, the sample was further processed in UV ozone cleaner (Novascan PDS-UV; Novascan Technologies, Inc., Ames, IA, USA) to remove any organic impurities on the Ni surface. The sample was then loaded into a homemade CVD furnace (Lindberg/Blue 1-in. diameter quartz tube; Thermo Fisher Scientific Inc., Waltham, MA, USA) at room temperature under Ar ambient with 200 standard cubic centimeter (sccm) flow rate. After ramping the temperature to 1,000°C, the sample was annealed in H_2_:Ar (65, 200 sccm) ambient for 10 min. BLG was then synthesized by flowing CH_4_:Ar (23, 200 sccm) for 2 min, and moving the hot portion of the tube to the room temperature by ultrafast cooling [13]. Research grade 5.0 (minimum purity 99.999%) process gasses supplied by Praxair Inc. (Danbury, CT, USA) were used. A 100 nm C_60_ film was deposited on the Ni/BLG structure, by using thermal evaporator (Edwards Coating System, E306A; Edwards Coating System, Sanborn, NY) at 1 Å/s rate under < 7 × 10^−7^ Torr chamber pressure. The commercial C_60_ powder was supplied by M.E.R Corporation (Tucson, AZ, USA). The use of C_60_ molecules ensures uniformity of the channel material constituents. Next, the sample was loaded in the electron-beam evaporator, and 5 nm of SiO_2_ was evaporated, followed by 90 nm of Cr by using a shadow mask. The evaporation rates of SiO_2_ and Cr were 0.2 and 1 Å/s, respectively, and the chamber pressure was < 1 × 10^−7^ Torr. A control sample without BLG was also fabricated as shown in Figure 1b. The thin layer of SiO_2_ was used to protect C_60_ film during subsequent metal evaporation step.

A detailed characterization of the synthesized BLG has been reported earlier in [13]. Raman spectroscopy was used to confirm the quality of evaporated C_60_. A laser power of 2 mW with 5 s scan time and four scans per point is used to avoid sample heating. The Raman spectrum of evaporated C_60_ film on BLG is also shown in Figure 1c. The dominant peaks are at 491, 1,464, and 1,596 cm^−1^ wavenumbers, which confirm the coherence of C_60_ molecular structure even after thermal evaporation [14,15].

## Results and discussion

**Figure 2:**
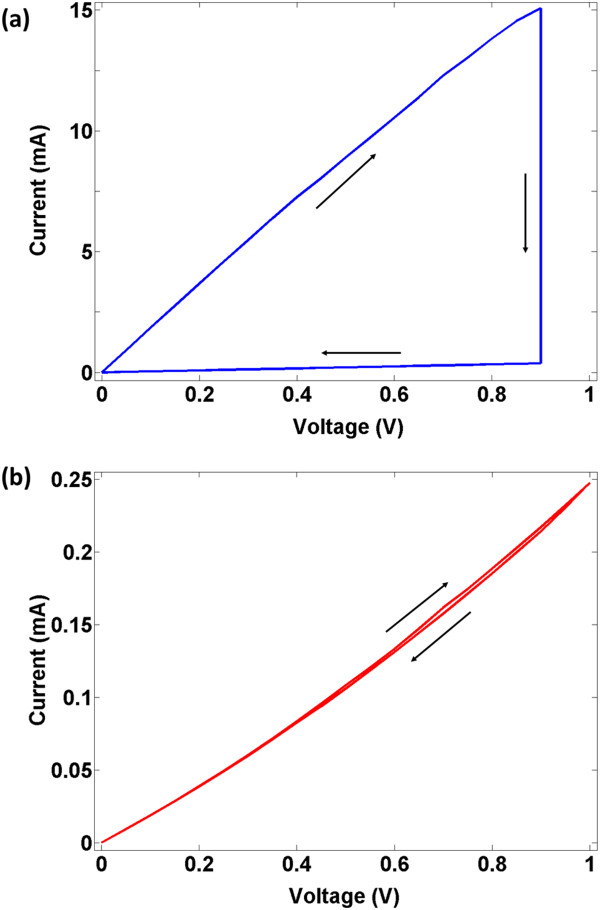
**Transport characteristics in the first and second sweep cycles. (a)** During the first sweep cycle, the voltage is swept in the forward direction until the device switches to high-resistance state. During the reverse sweep, the device remains in the high-resistance and shows hysteresis. **(b)** The device remains in the high-resistance state during the second sweep cycle and no hysteresis or switching is observed.

In Figure 2, we report the transport characteristics in the first and second sweep cycles for the device with BLG contact. The device starts in the low-resistance state and the voltage is increased in the forward direction until it irreversibly switches to high-resistance state at about 0.9 V, as shown in Figure 2a. After switching, the device withstands its high-resistance state, thus exhibiting hysteresis in the first cycle. We rule out the possibility of conductive filament formation (CFF) due to electromigration, since graphene has a breaking strength value of approximately 42 N/m and is impermeable even to helium atoms [16,17]. Moreover, in the CFF, current increases after switching, whereas an opposite trend is observed here. Apart from this, we find that the switching voltages for various devices lie in the 0.8 to 1.2 V bias range. This variation may be due to the amorphous and heterogeneous nature of the evaporated SiO_2_ film [18].

The switching behavior for the second sweep cycle is shown in Figure 2b. The device remains in the high-resistance state without hysteresis. In the subsequent sweep cycles, the device sustains its high-resistance state, thus making it a write-once read-many (WORM) memory device.

**Figure 3:**
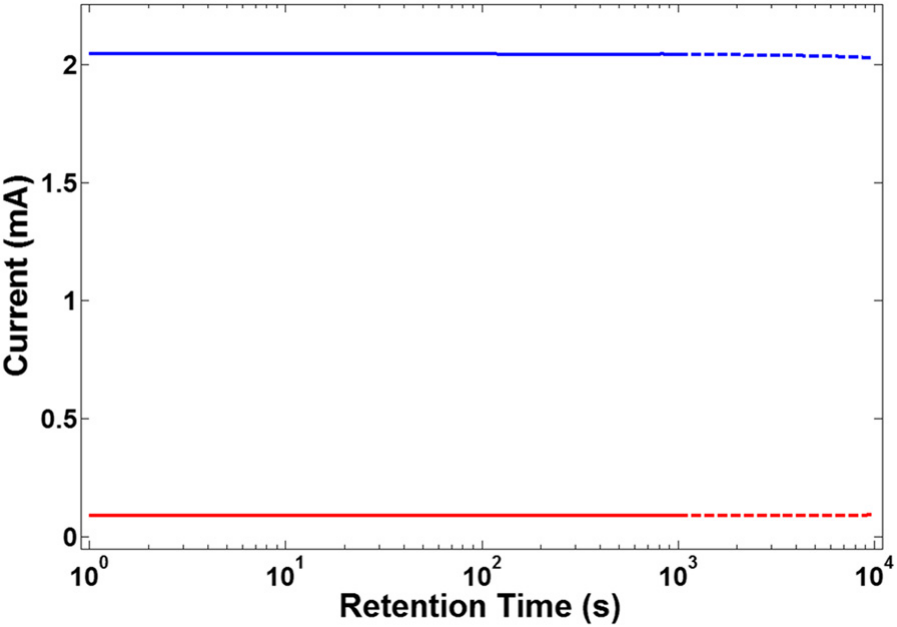
**Retention characteristics.** The memory device shows a stable low-resistance state with for 10^3^ s (blue line). After switching to the high-resistance state by applying a 1.2-V write pulse of 10 ms duration, stable current is observed again. The dashed lines are the interpolation to 10^4^ s (red line).

Next, we report the retention characteristics in Figure 3, by using a read voltage pulse train of 0.4 V bias with 10 ms duration and 0.1% duty cycle. The mean value of current in the low-resistance state is 2.041 mA with a standard deviation of 0.973 × 10^−3^. The device is then switched to the high-resistance state by applying a program voltage pulse of 1.2 V bias with 10 ms duration. The mean value of current is 89.29 μA with the standard deviation of 0.155. The current ratio of low-resistance to high-resistance state in this device is about 22.85 (which varied in 20 to 40 range for various devices). Besides the high retention time, the device also shows good endurance when continuous reading cycles with small pulse duration is applied. The retention characteristics are extrapolated to 10^4^ s, and a stable behavior is foreseen in both states of the device.

For the control sample without the BLG contact, the device shows higher conduction with random switching, hysteresis, and significant variation from device to device. We attribute this irregular behavior in our control sample to the atomically rough interface between Ni and C_60_, as well as the electromigration of Ni atoms across C_60_/Ni interface.

The switching mechanism in the reported WORM memory device with the BLG contact is not clearly understood yet. However, we hypothesize that BLG prevents the electromigration of Ni atoms into C_60_ film, thus stabilizing the device behavior. The transport characteristics do not show ohmic or space-charge-limited conduction. Similar devices using C_60_ molecules have been reported to have rewritable switching characteristics - quite different from our observation [19,20]. Moreover, multilayer graphene electrodes used in devices with PI:PCBM composite as active material have also been recently reported to have WORM memory behavior, whereas with the metallic electrodes, rewritable switching characteristics have been reported [21]. Although the channel materials are different in the two experiments, the connection between the use of graphene and WORM features is noteworthy and needs to be explored further. Carbon nanotube-based contact [22] has also been explored to eliminate electromigration, however, we believe that graphene nanomembrane provides a better interface due to its 2D nature.

## Conclusions

We have fabricated a molecular memory device with atomically smooth BLG contacts. Covering Ni film with BLG shields the channel from metal surface irregularities and also prevents the electromigration of Ni atoms into the C_60_ film. The device switches from a low-resistance to a high-resistance state, followed by hysteresis in the first sweep cycle. In the subsequent sweep cycles, the device remains in the high-resistance state and no hysteresis is observed, thus showing WORM memory behavior. The switching voltages vary in 0.8 to 1.2 V bias range for various devices with the high-resistance to low-resistance ratio in 20 to 40 range. The retention characteristics show good endurance under both low-resistance and high-resistance states up to 10^4^ s. In addition, replacing the top Cr/SiO_2_ contact with BLG may further improve the characteristics, which we leave for future work.

## Competing interests

The authors declare that they do not have any competing interests.

## Authors’ contributions

AU, TR and HR have equal contribution to this work and the manuscript. All authors read and approved the final manuscript.

## Authors’ information

AU received his B.Sc. degree in Electrical Engineering from the University of Engineering and Technology, Lahore, Pakistan, in 2007 and is currently working towards his Ph.D. degree in Electrical and Computer Engineering at the University of Iowa. His research interests include novel non-volatile memories, resistive random access memories, flash memories, and carbon nanomaterial synthesis.

TR received her B.Sc. honors in May 2001 from the University of Engineering and Technology Lahore, Pakistan majoring in electronics and communication engineering. Afterwards, she worked in Accelerated Technologies Inc. Pakistan, as a software engineer. She worked in SIEMENS Pakistan, for another year before she joined Purdue University, West Lafayette, IN, USA for Ph.D. program. She graduated from her Ph.D. in December 2010 and joined the University of Iowa, USA as adjunct Assistant Professor in the Department of Electrical and Computer Engineering and Department of Physics and Astronomy. Presently, she is an Assistant Professor at Lahore University of Management Sciences, Pakistan.

HR is a Professor of Electrical Engineering at the University of the Punjab, Lahore, Pakistan since 2012. Earlier, he was an Assistant Professor of Electrical and Computer Engineering at the University of Iowa, Iowa City, USA in 2009 to 2013. He was a postdoctoral associate at Cornell University in 2007 to 2009. He received his Ph.D. in 2007 and MS in 2002 from Purdue University; and B.Sc. in 2001 from the University of Engineering and Technology Lahore Pakistan. He has received ‘Magoon Award for Excellence in Teaching’ from Purdue University in 2004. He is also the recipient of ‘Presidential Faculty Fellowship’ in 2010 and ‘Old Gold Fellowship’ in 2011 from the University of Iowa. He has been awarded ‘Junior Associateship’ of the International Centre for Theoretical Physics, Trieste, Italy in 2013. His research group is focused on ‘anything that is small’ for low-power post-CMOS transistor, spintronics, sensors, and solid-state energy harvesting applications from theoretical, experimental, and computational approaches using graphene, molecule, silicon, novel dielectrics, and carbon nanotube material systems. He has served as an editor of a 600-page book on *Graphene Nanoelectronics* published by Springer in 2012.
